# Non-destructive Plant Biomass Monitoring With High Spatio-Temporal Resolution *via* Proximal RGB-D Imagery and End-to-End Deep Learning

**DOI:** 10.3389/fpls.2022.758818

**Published:** 2022-04-13

**Authors:** Nicolas Buxbaum, Johann Heinrich Lieth, Mason Earles

**Affiliations:** ^1^Department of Biological and Agricultural Engineering, University of California, Davis, Davis, CA, United States; ^2^Department of Plant Sciences, University of California, Davis, Davis, CA, United States; ^3^Department of Viticulture and Enology, University of California, Davis, Davis, CA, United States

**Keywords:** controlled environment agriculture, deep learning, biomass, monitoring, lettuce, computer vision, artificial intelligence, phenotyping algorithms

## Abstract

Plant breeders, scientists, and commercial producers commonly use growth rate as an integrated signal of crop productivity and stress. Plant growth monitoring is often done destructively *via* growth rate estimation by harvesting plants at different growth stages and simply weighing each individual plant. Within plant breeding and research applications, and more recently in commercial applications, non-destructive growth monitoring is done using computer vision to segment plants in images from the background, either in 2D or 3D, and relating these image-based features to destructive biomass measurements. Recent advancements in machine learning have improved image-based localization and detection of plants, but such techniques are not well suited to make biomass predictions when there is significant self-occlusion or occlusion from neighboring plants, such as those encountered under leafy green production in controlled environment agriculture. To enable prediction of plant biomass under occluded growing conditions, we develop an end-to-end deep learning approach that directly predicts lettuce plant biomass from color and depth image data as provided by a low cost and commercially available sensor. We test the performance of the proposed deep neural network for lettuce production, observing a mean prediction error of 7.3% on a comprehensive test dataset of 864 individuals and substantially outperforming previous work on plant biomass estimation. The modeling approach is robust to the busy and occluded scenes often found in commercial leafy green production and requires only measured mass values for training. We then demonstrate that this level of prediction accuracy allows for rapid, non-destructive detection of changes in biomass accumulation due to experimentally induced stress induction in as little as 2 days. Using this method growers may observe and react to changes in plant-environment interactions in near real time. Moreover, we expect that such a sensitive technique for non-destructive biomass estimation will enable novel research and breeding of improved productivity and yield in response to stress.

## Introduction

Plant growth is a foundational biological process that underlies both agricultural and ecological productivity. Biomass accumulation is the final product of photosynthetic CO_2_ assimilation and its rate is closely tied to traits such as productivity and stress response (Muchow, 1988; Scully and Wallace, 1990). Growth rate is linked to crop productivity and yield for grain, fruit, and vegetable crops. Vegetative crop growth rate, for example, is a strong predictor of final grain production in rice, soybean, wheat, and maize (Egli and Zhen-wen, 1991; Karimi and Siddique, 1991; Takai et al., 2006; Egli, 2019). Among leafy vegetables, growth rate is even more directly tied to yield as the leaves, and often stems, are harvested as the final product. Due to its close link to yield, growth rate is commonly measured in response to limitation and excess application of inputs such as light (Zhou et al., 2019), temperature (Zhou et al., 2019), nutrients (Sapkota et al., 2019), and water (Gallardo et al., 1996). Consequently, growth rate in response to variable inputs provides an optimization criterion for breeding higher input efficiency crops (e.g., nitrogen and water use efficiency; Zotarelli et al., 2009). Beyond breeding, growth rate monitoring provides commercial agricultural producers a means for detecting stress and understanding growth over time, both of which can lead to more precise planning and optimization of management practices (Kacira et al., 2005). Thus, plant growth monitoring is a critical tool for breeders, scientists, and commercial producers in their efforts to manage and develop more productive and stress tolerant crops.

The most direct method of determining plant biomass growth is *via* destructive sampling, which requires harvesting and weighing each individual (Catchpole and Wheeler, 1992). The destructive nature of this method reduces its utility in breeding and commercial settings as it often necessitates prohibitive numbers of individuals to generate the representative samples required for daily or sub-daily population biomass estimates. On the other hand, non-destructive biomass estimation allows for continuous measurement of individual plants which substantially reduces plant number requirements for effective experimentation and monitoring. Hand-gathered allometric methods that relate volume and height data to biomass are time-consuming, laborious, and may generalize poorly (Pottier and Jabot, 2017). The recent development of proximal and remote sensing-based approaches offers the promise of lower data acquisition cost and increased throughput and accuracy. Such methods generally involve computer vision-based analysis of color (Jung et al., 2015; Jiang et al., 2018) and 3D data modalities (Mortensen et al., 2018; Hu et al., 2018; Jin et al., 2020). Data is typically acquired from one or more viewpoints using color sensors, RGB-D cameras, or LiDAR systems. Then, plant pixels (or in the case of 3D data, voxels or 3D points) are segmented from the background *via* either classical or machine learning methods (Jung et al., 2015; Jiang et al., 2018; Mortensen et al., 2018; Loresco et al., 2019; Jin et al., 2020). The segmented data is used to generate features that can serve as predictors of biomass such as pixel counts (Jiang et al., 2018), 3D volume (Mortensen et al., 2018; Jin et al., 2020), plant height (Hu et al., 2018; Jin et al., 2020), projected leaf area (Mortensen et al., 2018; Jin et al., 2020), and other color and structural features (Hu et al., 2018; Jin et al., 2020). More recently, promising results have been achieved with deep learning methods which do not rely on initial scene segmentation, but instead estimate biomass by directly mapping input images to biomass (Zhang et al., 2020).

**Figure 1:**
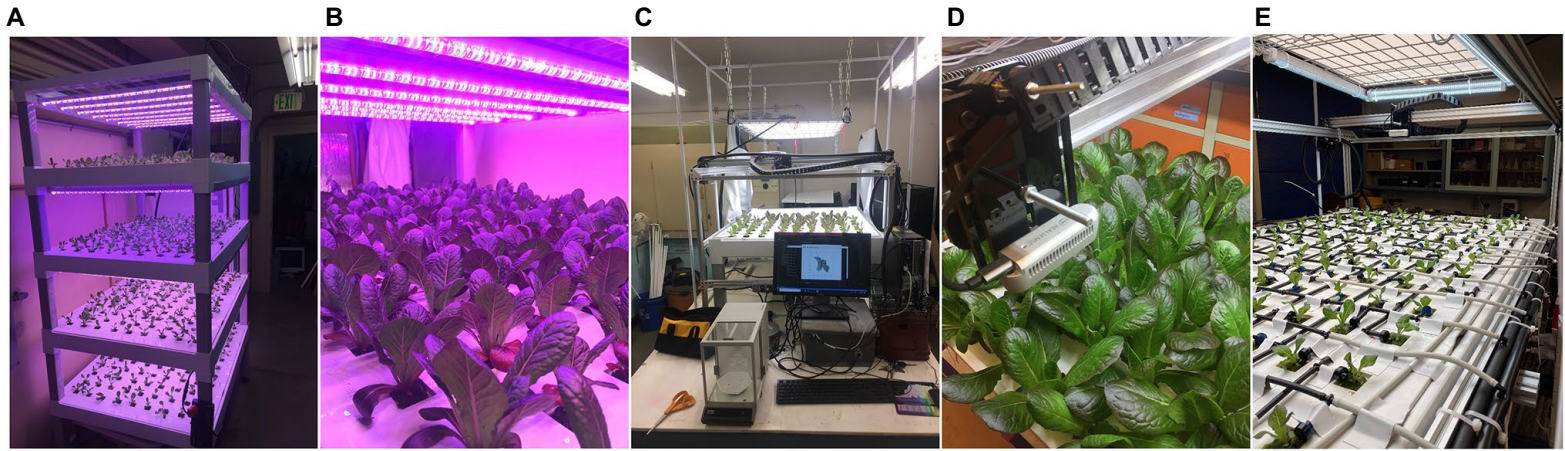
**(A,B)** The SananBio vertical cultivation system with individual lettuce plants at various growth stages. **(C,D)** The 2-axis imaging system equipped with an RGB-depth camera that was used to image lettuce plants and weigh destructively weigh individual plant mass values. **(E)** Our modified top fed drip system for growth monitoring under controlled nutrient stress.

Of these prior works, most rely on idealized scene conditions containing isolated individuals within field-of-view of the image. Only the methods of Jin et al. (2020) and Mortensen et al. (2018) are designed to estimate individual plant biomass within scenes containing the dense plant canopies typical of commercial agricultural settings. In commercial agriculture, high planting densities result in neighboring plants that create occlusions with each other, significantly increasing the complexity of the segmentation task. Furthermore, even with an effective segmentation algorithm, occlusions can cause large holes within the resulting segmented plant pixels, potentially reducing the accuracy of calculated biomass predictors. These problems are greatly exacerbated in leafy green production, where canopies can become near continuous (Figures 1A–C). While both works developed effective plant-from-plant segmentation schemes, neither tested their methods on extremely high density continuous canopies (Mortensen et al., 2018; Jin et al., 2020). Further, both methods rely to some degree on human input, greatly reducing the throughput and advantage of remote sensing based biomass estimation.

To solve the challenge of accurate and autonomous individual plant biomass estimation within high density canopies, we propose an end-to-end deep learning approach. Our end-to-end approach eliminates the need to perform explicit individual plant segmentation and instead allows a deep convolutional neural network (DCNN) to implicitly perform segmentation by learning a mapping from input image space to individual plant biomass. Motivated by previous biomass estimation work that relies on 3D data as well as the ability of DCNNs to jointly learn from color and depth imagery (Gupta et al., 2014; Eitel et al., 2015; Ophoff et al., 2019; Ward et al., 2019), our model incorporates both color and depth data as provided by an inexpensive and commercially available stereovision RGB-D camera. We hypothesize that DCNNs are well suited to understanding not only plant structure and size, but the influence of neighboring plant occlusion on the resulting view presented in overhead imagery of dense plant canopies.

In this work we develop a novel approach to non-destructively monitor crops by estimating the fresh biomass of individual lettuce plants grown in a typical commercial hydroponic cropping system from a proximal overhead viewpoint. By combining RGB-D sensing with a deep learning regression approach, we demonstrate state-of-the-art performance for quantifying biomass across the entire range of the lettuce growth cycle, from transplant (< 1 g) through maturity (> 30 g). Our approach and results not only have implications for leaf lettuce but can more broadly be applied to estimate biomass and other phenotypic traits of crops grown in occluded environments. To validate our model’s ability to function as a crop monitoring system, we perform an additional experiment which subjects plants to various heterogeneously applied nutrient stresses. This rigorously tests the model’s ability to capture varying trends in growth between neighboring individuals and shows that monitoring growth dynamics at the individual plant scale is possible under non-idealized cropping conditions.

## Materials and Methods

### Dataset for Deep Learning Model Creation

Our dataset used in model creation is composed of pairs of overhead images and biomass values of the baby leaf lettuce cultivar *Lactuca sativa* var. Powerhouse grown in a high density hydroponic cropping system. Every image was centered upon a unique individual lettuce plant and the associated biomass value was the measured fresh above ground biomass of the corresponding center plant in the image.

#### Cultivation System

Plants used in this work were grown indoors within a laboratory facility at the University of California, Davis campus. Plants were grown in continuously recirculating nutrient film technique hydroponic systems (SananBio, Xiamen, Fujian, China; Figure 1A). The systems were composed of four vertical tiers, with each tier holding two growing trays. One growing tray contained 54 plants arranged in a nine by five grid pattern with an approximate spacing of three inches. Seeds were germinated in one-inch rockwool cubes and transplanted into the SananBio system growing trays after first emergence of true leaves. Plants were grown until imaging and destructive measurement of fresh biomass was performed. Each rockwool cube contains the plant’s root system which is free to grow beyond the cube into a drainage channel. Photosynthetic photon flux was maintained continuously at an average of 135 μmol·s^−^1·m^−2^ over the canopy area. Temperature was maintained between 72 and 85°F.

#### Imaging System

The chosen imaging system utilized an Intel RealSense d435i (Intel Corp., Santa Clara, CA, United States) camera mounted vertically over the imaging bay at a height of 37 cm (Figures 1C,D). It was actuated to positions directly above each plant by a stepper motor and belt driven positioning system. While the theoretical tolerances of the positioning system are on the order of 1 mm, inaccuracies due to deformations of the plastic trays and alignment with the imaging system resulted in observed positional tolerances of approximately 1 cm. Images were always taken while the camera was not in motion. Data collected consisted of an 848 pixel by 480 pixel 8-bit color RGB image as well as an associated depth image. The depth image is a 2D image, where each pixel represents the distance between the sensor and the in-scene object with a precision of 0.1 mm. No filtering was applied to the depth images. We chose to utilize a depth to color image alignment scheme with the Intel RealSense 435i, which involves a transformation of the depth image centered at one monochromatic sensor origin to the designated color sensor origin. The result was a depth image aligned to the color image (i.e., they share a coordinate system).

#### Data Collection

Data collection was performed on single trays, which correspond to a 54 plant subsample of the growth trial. During each data collection event a tray was removed from the cultivation system and placed under the imaging system. The illumination within the imaging system was maintained at approximately 100 lux and 6,500 K. Immediately after imaging, fresh above ground biomass was recorded. This was accomplished by severing the plants directly below the cotyledon and weighing them to a precision of 0.001 g or 0.1 g, depending upon the plant’s size and ability to fit within an analytical balance chamber. Each data collection event therefore yielded 54 RGB images, 54 aligned depth images, and 54 plant biomass values.

#### Growth Trials for Destructive Biomass Measurements

Six growth cycles were conducted sequentially over a 5 months period. Each cycle featured a varying number of total trays and plant count, although individual trays always contained a full 54 plants. During each growth cycle, data collection events started 1 week after transplant at a rate of three to four events per week and continued until all trays were harvested. This resulted in plants with a harvest date between 7–30 days after transplant. A total of 3,888 plants were harvested and their biomass values destructively measured.

### Non-destructive Growth Monitoring in Response to Stress Induction

After achieving satisfactory biomass prediction accuracy, we implemented the model in a plant monitoring application to track growth over time from transplant to harvest. We modified our previous hydroponic system from nutrient film technique, where every plant received the same fertigation solution, to a top-fed drain to waste system (Figure 1E). This allows for the application of unique fertigation solution to four subsets of plants in a random spatial arrangement. Each plant site was fertigated by a single 0.5 gallon per hour pressure compensating emitter for 1 min every 7 h. Four separate pumps supply the system, each connected to drip lines arranged in an alternating pattern along each axis. By supplying each pump with a certain fertigation solution, we can dynamically select the fertigation solution experienced by any particular subset of the plants. The entire growing system was canted to facilitate draining and reduce the uptake of shared leachate by plant roots. We maintain equivalent lighting and transplanting conditions as the original dataset.

#### Experimental Design

Our experiment consisted of 108 plants that were completely randomly distributed into four groups of 27 plants (*n* = 27; Figure 1E). Each group was subjected to a different schedule of nutrient stress inductions and reductions designed to dynamically modify the growth rate of each treatment group over time (Table 1). Since the plants were grown hydroponically, nutrient stress was induced by providing pure distilled water through the irrigation system. To return to non-stress conditions, fertigation was resumed with half strength Hoagland solution. We composed a schedule of nutrient stress applications for each of three treatments and a control (Table 1). For each treatment we indicate the number of days after transplant (rounded to the nearest imaging date) that stress conditions were induced or reduced. All treatments started with Hoagland solution after transplant (Day 0). The “control” treatment receives no stress induction and is provided Hoagland solution for the entire growth period.

**Table 1:** Treatment schedules as begun on particular days (“Solution” = half strength Hoagland solution; “Water” = De-ionized distilled water).

Treatment	*n*	Day 0	Day 6	Day 11.7	Day 15.7	Day 15.7
A	27	Solution	Water	Solution	Solution	Solution
B	27	Solution	Solution	Water	Water	Solution
C	27	Solution	Solution	Water	Solution	Water
D (control)	27	Solution	Solution	Solution	Solution	Solution

#### Data Collection

We utilized the same imaging system as in the original model training dataset. The only modification made was within our software to allow for automated image capture according to a set interval. Images were taken every 8 h for the duration of the experiment, with each imaging event consisting of 108 images. This resulted in a sequential representation of plant growth composed of 96 images per plant over the 32-day experiment (a total of 10, 368 sets of RGB and depth images). Imaging of the entire tray of 108 plants required 5 min to complete.

In addition to the collected image data, we measure per-plant width (defined as the largest horizontal extent of the plant) and height (distance from the top of the growth tray to the tallest portion of the plant) at three separate dates (day 12, 16, and 23 from transplant). These non-destructive measurements provide us with some understanding of ground-truth plant growth, helping verify that our stress treatments truly induce the changes in plant growth that our model illustrates.

#### Model Detection of Stress Treatments

As the purpose of this work is to evaluate the suitability of a deep learning-based biomass estimation model as a crop monitoring technique, we evaluate how well the model predictions can capture changes in growth induced by controlled nutrient stresses applied heterogeneously over time and space. We approach this by examining the per-treatment model predicted mass, as well as the derived metrics growth rate (GR) and relative growth rate (RGR; Equation 1). For each metric we test for treatment effects by determining when the per-treatment means become statistically separable from each other over time. Based on our understanding of expected treatment effects, we can utilize the results of our statistical tests to determine which growth metrics are the most responsive determinants of nutrient stress.

Our test consists of applying Tukey’s HSD test (Tukey, 1949) for multiple pairwise comparisons to determine whether the treatment means are distinguishable at *p* ≤ 0.05 between any two treatments. We calculate this for each growth trait across the entire time series. RGR is calculated for each plant within a given data collection event by first applying a backwards looking moving-average with a window size of 3, followed by a gradient based RGR calculation given by Equation 1.


(1) $$RGR_{t}=\frac{GR}{m_{t}}\text{ with }GR=\frac{m_{t}-m_{t-1}}{\Delta t}$$

where m_t_ = moving average biomass at time *t* and Δ*t* = difference in time between *t* and *t*-1 (units are 8 h). Boundary cases in the moving average and RGR calculations are dropped.

### RGB-D Regression Network Architecture

**Figure 2:**
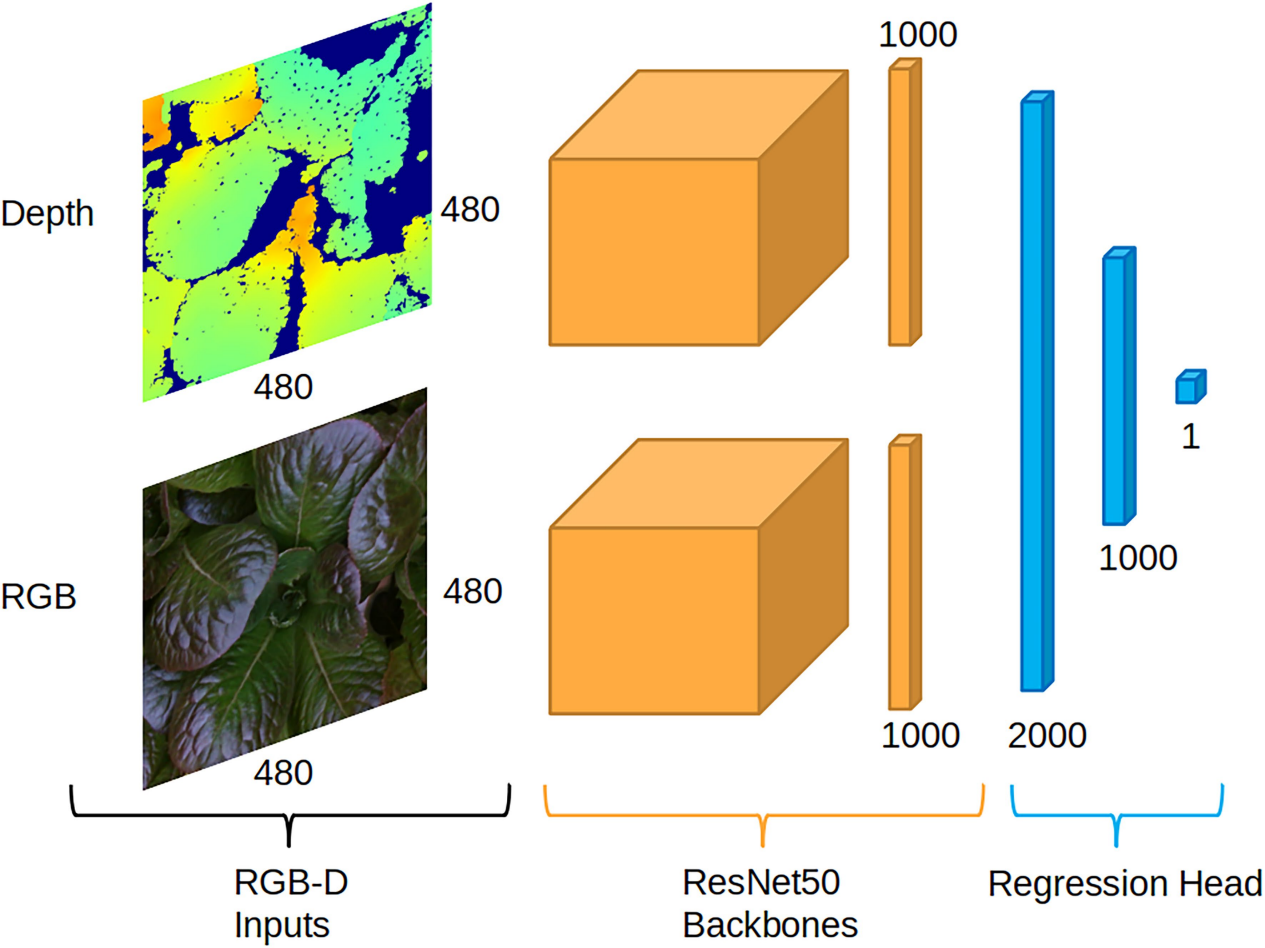
The late fusion multimodal regression network. Depth and RGB data are processed by two separate ResNet50 branches. The output of each branch, a 1,000 × 1 layer, are concatenated and then used as input into the regression head, whose final layer produces the biomass prediction.

Our choice of architecture is modeled from successful work in RGB-D object detection and classification utilizing feedforward neural networks (Eitel et al., 2015; Ward et al., 2019). We utilize a dual-branch architecture for each input data modality along with feature map fusion (Figure 2). In particular, Ophoff et al. (2019) empirically demonstrated that a mid to late fusion architecture performs best for real-time object detection when utilizing YOLOv2 as the feature extraction network for each branch. We utilize ResNet-50 for feature extraction and retain the final 1,000 × 1 fully connected layer from ImageNet’s 1,000 classes while removing the softmax activation function. The fully connected layer from each branch is then end to end concatenated into a feature vector of 2000 × 1. This feature vector is passed to the regression head, which consists of one fully connected 1,000 × 1 layer and one 1 × 1 layer. Rectified linear unit functions are utilized as the activation function between all layers besides the final, which is simply a linear activation. In testing we found no benefit to increased depth of the regression head. The resulting late fusion network can leverage transfer learning with ImageNet pretrained weights for fast convergence with our relatively small dataset (Figure 2).

#### Model Loss Function

We train the network to minimize a mean average percent error (MAPE) loss function (Equation 2). While known to produce biased under estimations in statistical prediction problems, in our testing an MAPE loss function resulted in more even model performance across the entire range of possible plant sizes than either L1 or L2 loss functions. We speculate that this is due to the MAPE loss function driving model learning as a function of relative, rather than absolute, difference between the model predictions and ground truth. This phenomenon is especially pronounced in our dataset, which features a range of plant mass values in the order of 300x—from 0.1 g to more than 30 g. It should be noted that we favor relative error and consistent model performance across all plant sizes as it better pertains to our goal of utilizing plant mass estimation models to inform crop status across the entire growth cycle.


(2) $$MAPE=\left(\frac{1}{n}\overset{n}{\underset{i=1}{\sum }}\left|\frac{A_{i}-F_{i}}{A_{i}}\right|\right)$$

where *n* = number of samples, A_i_ = i-th sample ground truth mass, and F_i_ = i-th predicted mass.

#### Model Input Preprocessing

While our color data may be utilized directly by the RGB branch of the model, the single channel depth data lacks the 3-channel dimension expected by the first layer of the pre-trained Resnet50 depth branch. Possible solutions of adapting non 3-channel input data for use with RGB pre-trained networks generally fall into two distinct pre-processing schemes: (1) Expanding the third dimension of the input data to 3 channels or (2) replacing the first layer of the network with a new convolutional kernel that expects a different size channel dimension. The former solution allows for full retention of pre-trained weights, while the second requires a new, untrained weight initialization for the first convolutional layer. We find that encoding of the single channel depth input to three channel RGB, as proposed by Eitel et al. (2015), results in improved learning compared with other pre-processing schemes tested: (1) single channel depth combined with a new, randomly initialized first convolutional layer and (2) three times replicated depth channel input with pretrained first convolutional layer. We follow a “jet” color scheme that maps the depth output of the RealSense camera to RGB color. The depth and corresponding color images are linearly normalized to [0, 1] and maintain their precision *via* a 32-bit floating-point number. Normalization is achieved by first filtering out large depth data values corresponding to the non-plant scene, defined as any depth value larger than 380 mm (a number arrived at by taking into account the camera field of view and height from the growing tray). This reduces the range of depth values from [0, 12,000] (the maximum distance possible in the scene) to [0, 3,800] while maintaining all relevant scene information. Reducing the depth data range increases the contrast of the resulting RGB mapping. Finally, both color and depth images are center cropped to 480 by 480 pixels.

#### Model Training Details

The model is implemented using the PyTorch framework within Python (Paszke et al., 2019). Each branch of the RGBD network is initialized from modality-specific pre-trained weights. This is achieved by first training each branch separately as its own model to predict lettuce biomass. Those branches are initialized *via* ImageNet pre-trained weights available from the PyTorch’s model zoo and are trained with a regression head consisting of a single 1000×1 fully connected layer. The regression head is then removed and the remaining branch weights used to initialize the weights for final fine-tuning of the RGBD model. All relevant training parameters are kept consistent across the RGB, depth, and RGBD networks.

**Figure 3:**
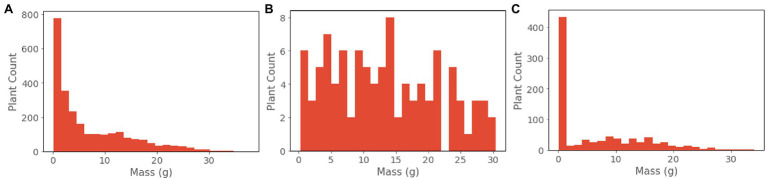
**(A)** Training, **(B)** validation, and **(C)** test data distributions.

The training dataset is composed of 2,484 plants, corresponding to the remaining data from the validation and test sets as described in Section 2.4. (Figure 3**A**) The weights and biases of the network are learned using the AdamW optimizer with a weight decay of 0.001. The learning rate is set to 0.0001 and is decreased by 50 percent at epochs 20, 40, 60, 80, and 100. We use a mini batch size of 16 on a single Nvidia Titan RTX GPU. Training is terminated at convergence, defined as when no decrease in epoch validation loss is observed for 60 continuous epochs.

#### Training Data Augmentation

We apply regularization by utilizing several problem and data appropriate data augmentation schemes at train time; all of which were done using PyTorch Torchvision augmentation functions (Paszke et al., 2019). The first is a random, image center-based rotation of both depth and color images from 0 to 360 degrees. We speculate that this is an effective augmentation strategy that preserves the input data distribution due to the symmetrical nature of lettuce plant growth combined with the location of the plant apex at the image center. The second is a random manipulation of brightness, contrast, saturation, and hue by a factor of 0.5–1.5. The third is a grayscale transformation of the color image applied with a probability of 0.1. The second and third augmentation schemes are applied to the color input image only and serve to reduce model dependency on color. The color images in our dataset are not of uniform quality due to unaccounted for changes in scene brightness relative to camera exposure time. We choose not to apply similar augmentation schemes to the depth data, as the mapped RGB channel distribution and intensity values are directly related to scene geometry as a result of their encoding from depth information.

### Model Selection and Evaluation

For the purpose of model selection, we created a validation set that better represents all possible plant conditions than the overall dataset (Figure 3B). This involved sampling from the overall dataset such that the validation set achieved a more, although not perfectly, uniform distribution across biomass values. We selected eight trays (378 plants) that represent harvest times from 14 to 35 days since germination. From this distribution, we subsample 100 individual data points *via* a weighted sampling scheme: biomass values are discretized into 35 bins corresponding to 1 g increments, and then are sampled by weighing each bin inversely to its count. Best performing models were selected based upon the lowest loss achieved on the validation dataset at model convergence.

Model evaluation criteria is an important factor for determining model performance in regression tasks. Common criterion for biomass estimates include MAPE (Equation 2) and root mean squared error (RMSE; Equation 3). However, for the problem of plant mass estimation, single value summary statistics give incomplete insight into model performance as they mask potential heteroscedasticity as well as imbalances in dataset distribution. The nature of our problem changes significantly over the range of biomass values and corresponding stage of development of the plants in the scene. Plant structure and size varies significantly over time, while larger neighboring plants introduce increasing occlusions. Additionally, the end user of such a model may have varying use cases and accuracy requirements.


(3) $$RMSE=\sqrt{\frac{1}{n}\overset{n}{\underset{i=1}{\sum }}{\left(A_{i}-F_{i}\right)}^{2}}$$

Where *n* = number of samples, A_i_ = i-th sample ground truth mass, and F_i_ = i-th predicted mass.

For these reasons, during model evaluation we report RMSE and MAPE on a per-group basis for ranges of ground truth mass values. These groups were chosen based upon the relative levels of occlusion experienced by plants of those mass values on average. We present the results of all models evaluated on the unseen test dataset. Our test set was composed of 864 plants sourced from a single growth cycle not present in either the training or validation sets (Figure 3C). Sourcing all test data from a separate growth cycle reduces the possibility of overfitting to variations in phenotypic expression within the different growth trials.

### Model Ablation Study

The rationale for utilizing joint RGB and depth data modalities as predictor variables in a biomass estimation model centered on three facets: (1) the current state of the art detection and semantic segmentation models utilize RGB data, (2) many current biomass estimation methods utilize 3D representations, and (3) DCNNs can effectively learn jointly from each modality. However, we find it worthwhile to further investigate whether the combined data modalities truly result in superior predictive performance over either modality individually. We conduct an ablation study which tests the ability of each data modality to predict plant biomass. In this study, we present the predictive results of the RGB and depth only networks that also form the weights used during the initialization of the RGBD network. Each network mirrors the RGB-D regression network, with identical training parameters as well as input preprocessing, pretraining, and similar regression head.

### Model Interpretation *via* Gradient Class Activation Mapping

Intuitively, the problem of individual plant biomass estimation requires an understanding of the extent and physical characteristics of the plant of interest in the scene, including an approximation of any occluded or out of scene portions. Due to the nature of our loss function, there is no strict enforcement of localization, leaving us unsure as to whether the model is in fact learning center plant mass, or simply some other mapping of common scene features of the dataset to biomass. This limits our trust in the model—and its broader applicability to problems involving individual plant mass estimation—by potentially reducing its generalizability outside of the dataset distribution. Due to our homogenous environmental conditions, our dataset generally features scenes of plants of similar size and age. An ability to generalize outside of this distribution becomes important when considering certain applications of the model, such as growth rate abnormality detection where estimation of unlike neighbors may become important. While one could answer the question of generalizability through extensive testing within a target distribution, we forgo that additional expense and explore the question instead by investigating model decision making and predictive behavior. In particular, we utilize Gradient Class Activation Mapping (GradCAM; Selvaraju et al., 2016) to explore model localization *via* the latent space as well as examine examples of the success and failure modes of the model. GradCAM is a generalization of the class activation map method (Zhou et al., 2015) that allows for visualization of important latent space features in pixel space. We apply GradCAM to the final block of the RGB Resnet50 branch of our RGBD network, ignoring the depth branch contribution in favor of simplicity in understanding model localization. We report the visualizations of Guided GradCAM for the top five best and worst predictions over four different binned ranges of plant mass.

In addition to GradCAM, we explore the model prediction success and failure modes by visually examining the best and worst predictions. We hypothesize that a model that has truly learned center plant mass is likely to succeed and fail under different scene conditions than a model that has learned an unrelated mapping. For instance, the former might succeed when the center plant is less occluded, while the latter might succeed under scene conditions where plants are relatively similar (i.e., median of dataset distribution). Similarly, failure modes are likely to differ, such as when the center plant is under heavy occlusion, or when the center plant is of greatly different size than its neighbors (dataset distribution outlier). While this analysis is speculative in nature, we found it worthwhile to publish some best and worst case examples that may help illustrate what mapping the model has learned.

## Results

### Lettuce Image and Biomass Dataset

**Figure 4:**
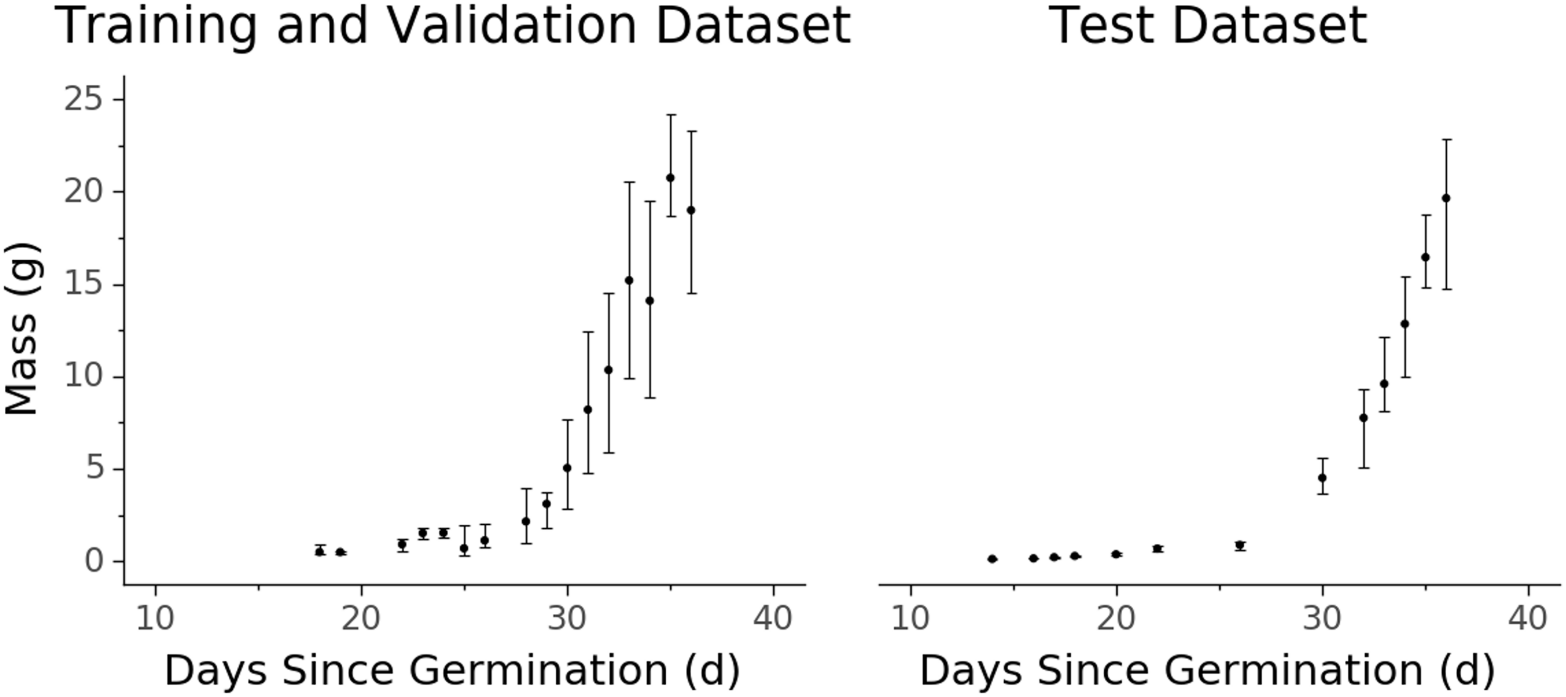
Biomass accumulation over time for the **(A)** training and validation and **(B)** test datasets measured destructively. Each data point represents one or more samples harvested at a certain age since germination. Points represent the median value while vertical extent bars indicate 25–75% quantiles.

The resulting dataset contains comprehensive representations of the lettuce cultivar ‘Powerhouse’ over its entire life cycle, totaling 3,888 RGB images, 3,888 aligned depth images, and 3,888 plant biomass values (Figure 4). Due to its size, a variety of phenotypic expressions are present, including variations in leaf color from green to dark purple. Some trays also exhibited stem elongation, likely a physical response to either shade condition or low air temperature.

There are some qualities of the dataset that are worth noting. Due to the exponential nature of plant growth, the dataset distribution is skewed towards smaller and younger plants (Figures 3, 4). Additionally, the plants were grown under well-watered and adequate nutritional conditions, and therefore the dataset contains no representations of plants under water stress or severe nutritional stress. A variety of possible environmental parameters exist that could result in phenotypic responses that are not represented in the dataset.

### Lettuce Image Data

**Figure 5:**
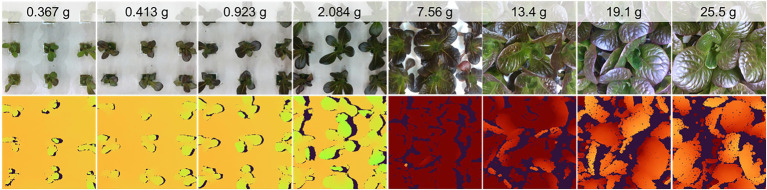
Examples of the color image data collected (at native resolution) and scene composition for plants at various ages and mass.

In their native resolution of 848 by 480, the images contain up to 15 plant sites, although this is reduced to nine plant sites at a resized 480 by 480 resolution for model use (Figure 5). At larger plant sizes, the scene changes significantly, with far fewer plants represented. In fact, the center plant’s full extent is often not completely represented in the resized images (Figure 5).

The color data were well focused and generally of high quality, although issues related to the RealSense camera’s automatic exposure algorithm resulted in some over- (low contrast, larger average pixel values) and under- (low contrast, smaller average pixel values) exposed images. Depth images contained some missing pixels due to errors in stereo matching due to occlusion and specular effects. The extent of missing data was generally greater for larger plants (Figure 5). The depth data were not evaluated for absolute measurement accuracy, although visualizations of the depth data show good representation of the scene despite missing data.

### RGBD Model

**Figure 6:**
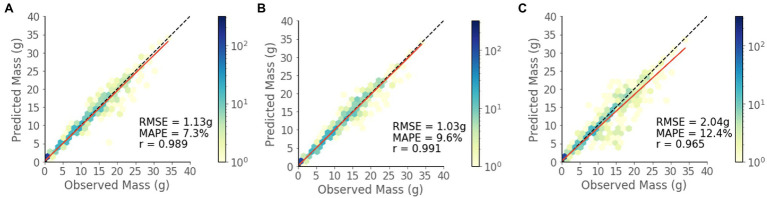
Predicted vs. observed mass values for the test set for each model. **(A)** RGBD, **(B)** RGB, and **(C)** Depth.

**Figure 7:**
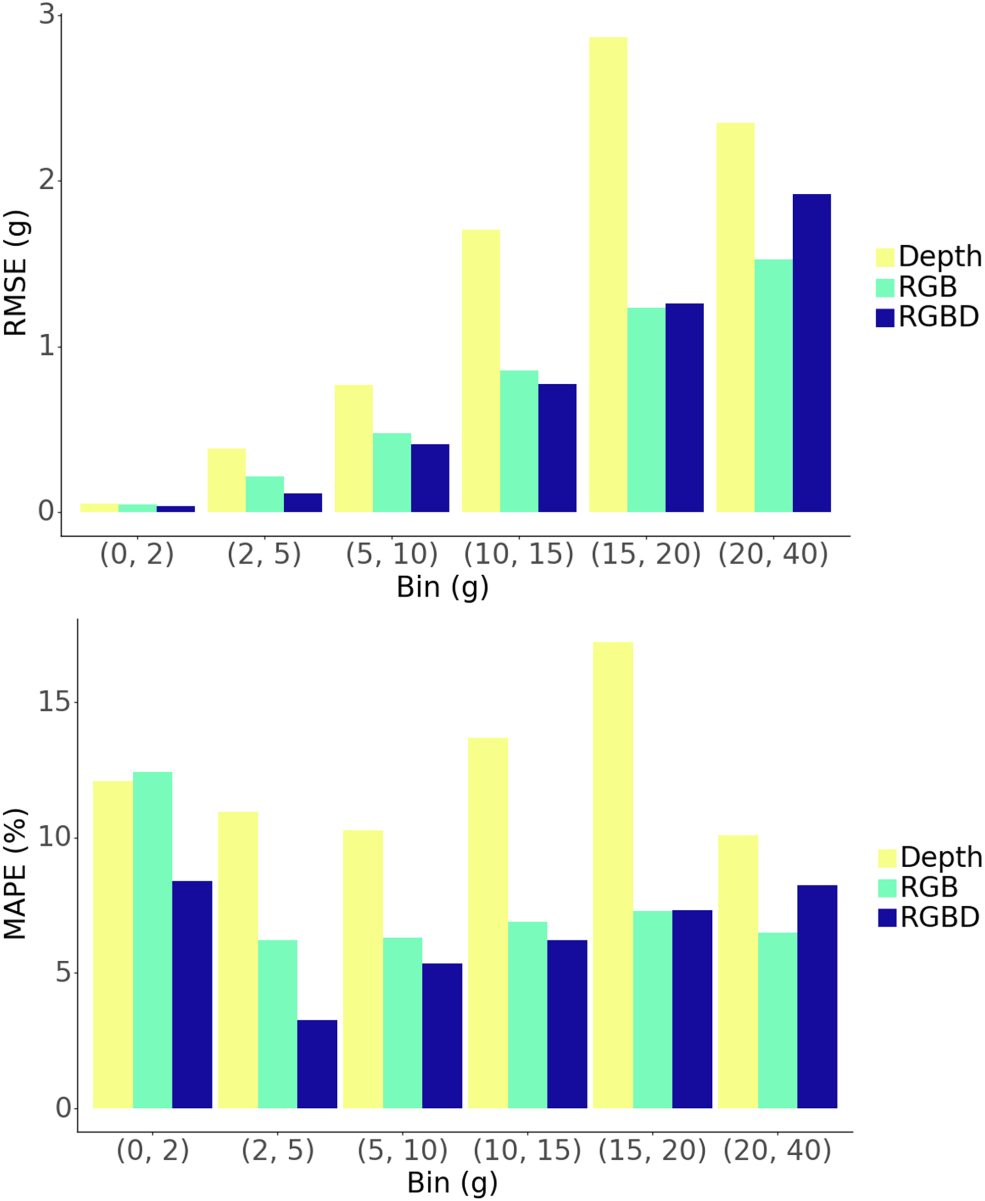
Binned evaluation metrics for the test set.

The model shows strong predictive performance over the entire 854 plant test set, achieving an RMSE of 1.13 g, a MAPE of 7.3%, and a Pearson’s correlation coefficient of 0.989 (Figure 6A). Some heteroscedasticity can be observed, and the greatest average relative errors occurred on the smallest and largest of plant masses, in addition to some variability in predictive power between that ranges (Figure 7).

### Model Data Modality Comparison

The RGBD model outperforms both RGB and depth only models in MAPE while the RGB model outperforms in RMSE (Figures 6B,C). Among the smallest plants, RGBD significantly outperforms each modality separately while the RGB and depth models achieve very similar relative and absolute error (Figure 7). As the plants increase in mass, the RGBD model generally outperforms the RGB and depth models while RGB outperforms depth. However, for plants between 10–15 g and 15–25 g, RGB shows comparable performance and even outperforms the combined RGBD modality in both MAPE and RMSE metrics.

### Model Prediction Performance Examination

**Figure 8:**
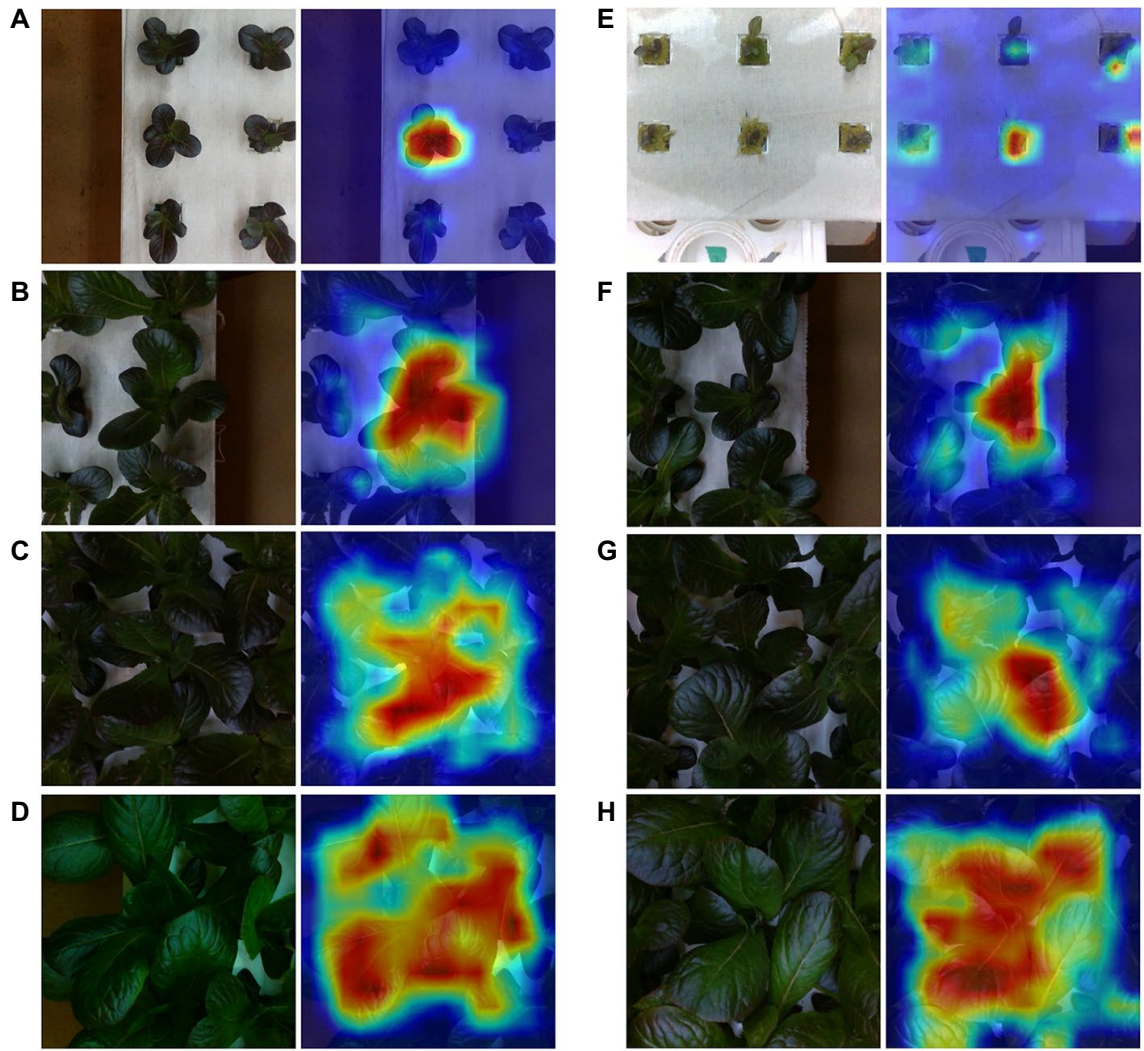
Sample best **(A–D)** and worst **(E–H)** predictions from the test set along with GradCam output for varying mass ranges. Each represents a mass range. Starting from the top are [0, 2] **(A,E)**, [2, 5] **(B,F)**, [5, 10] **(C,G)**, and [10, 25] **(D,H)**.

Among the best predictions, the model shows strong localization of the center plant (Figure 8). This tends to hold true across plant sizes, with important activations indicated along occlusion boundaries for larger plants. We notice a similar outcome among the worst predictions, although some cases show activations that are not centered on the center plant or appear to contain additional activations of neighboring plants that are not part of an occlusion boundary.

Examining the best and worst case model predictions reveals a nuanced relationship between prediction error and scene composition. We notice that in comparison to the high error predictions, low error predictions generally involve less occluded scenes. However, some examples of high error scenes show similar levels of occlusion to their low error counterparts, especially among examples of bins [2, 5] (Figure 8). In this bin, the worst performing cases do not appear contain stronger occlusion, but rather the center plant is significantly smaller than its neighbors.

### Model Predicted Biomass Response to Stress Treatments

**Figure 9:**
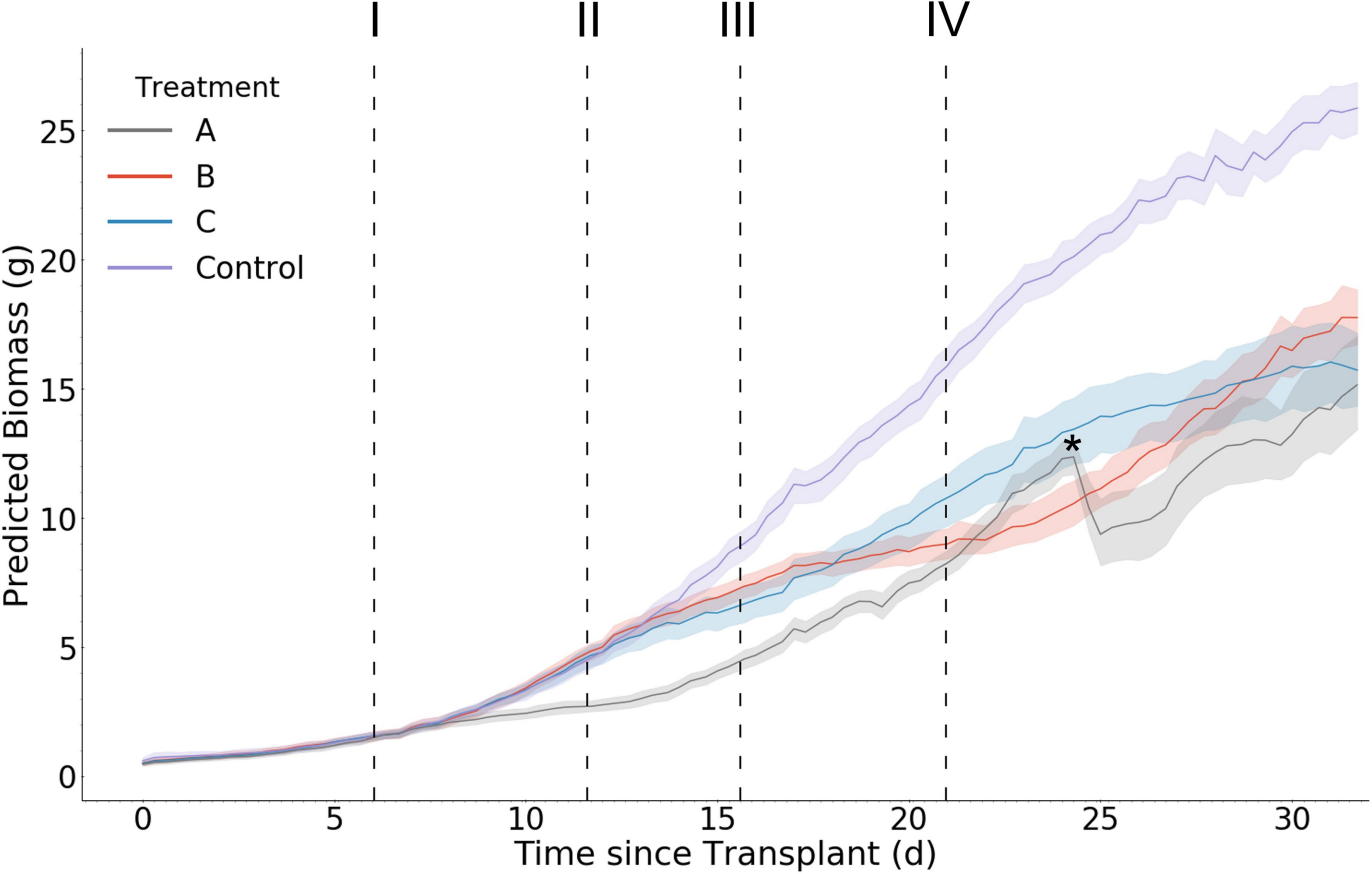
Mean per treatment predicted biomass with 95% confidence interval. Dark vertical lines and roman numerals indicate when treatments were applied. (I) Treatment A begins to receive pure water, (II) Treatment A begins to receive Hoagland solution and Treatments B and C begin to receive water, (III) Treatment C begins to receive Hoagland solution, and (IV) Treatment B begins to receive Hoagland solution and Treatment C begins to receive water. An asterisk marks where wilting in Treatment A was visually noticed in the image data (day 24.3).

The control (Treatment D) exhibits an expected sigmoidal biomass accumulation pattern approaching an asymptote after day 30 (Figure 9). All other treatments resulted in less biomass accumulation over the experimental period compared with the control.

Starting after the stress induction of treatment A on day 6, the mean biomass accumulation decreases. The mean separation (as calculated *via* Tukey HSD test) between treatment A and the other treatments occurs on day 9.3, approximately 3.3 days after the stress induction. No mean separation occurs before this day. At day 11.7, treatment A begins to receive nutrient solution, removing the nutrient stress and increasing biomass accumulation until day 24.3. Here, a malfunction of the irrigation system caused extensive wilting in some of the individuals within the treatment. This was remediated on day 26, and recovery can be seen at that time in the wilted plants (Supplementary Figure S1), as well is in the biomass predictions (Figure 9). After this recovery, the confidence interval associated with treatment A mean biomass estimation increases dramatically.

The response of biomass accumulation to the nutrient stress applied simultaneously to treatments B and C on day 11.7 is evident by their decrease in biomass accumulation compared with treatment D (control). There is a delay in the separation of the means between treatments C and D of 2.3 days after stress induction (day 14), and of 3 days between treatments B and D (day 14.7). No mean separation between these treatments occurs before day 14.

The response to the nutrient stress reduction applied to treatment C on day 15.7 is evident in the increase of biomass accumulation of treatment C compared with treatment B (Figure 9). Treatment C’s mean biomass becomes significantly larger than treatment B’s on day 20.7 (5 days after stress reduction).

The final scheduled stress event occurs on day 21, when treatment C begins to receive water and treatment B receives Hoagland solution. This results in the treatment means becoming inseparable at day 26, where it remains as such for the remaining duration of the trial.

### Model Predicted Growth Rate and Relative Growth Rate as Stress Response Indicators

**Figure 10:**
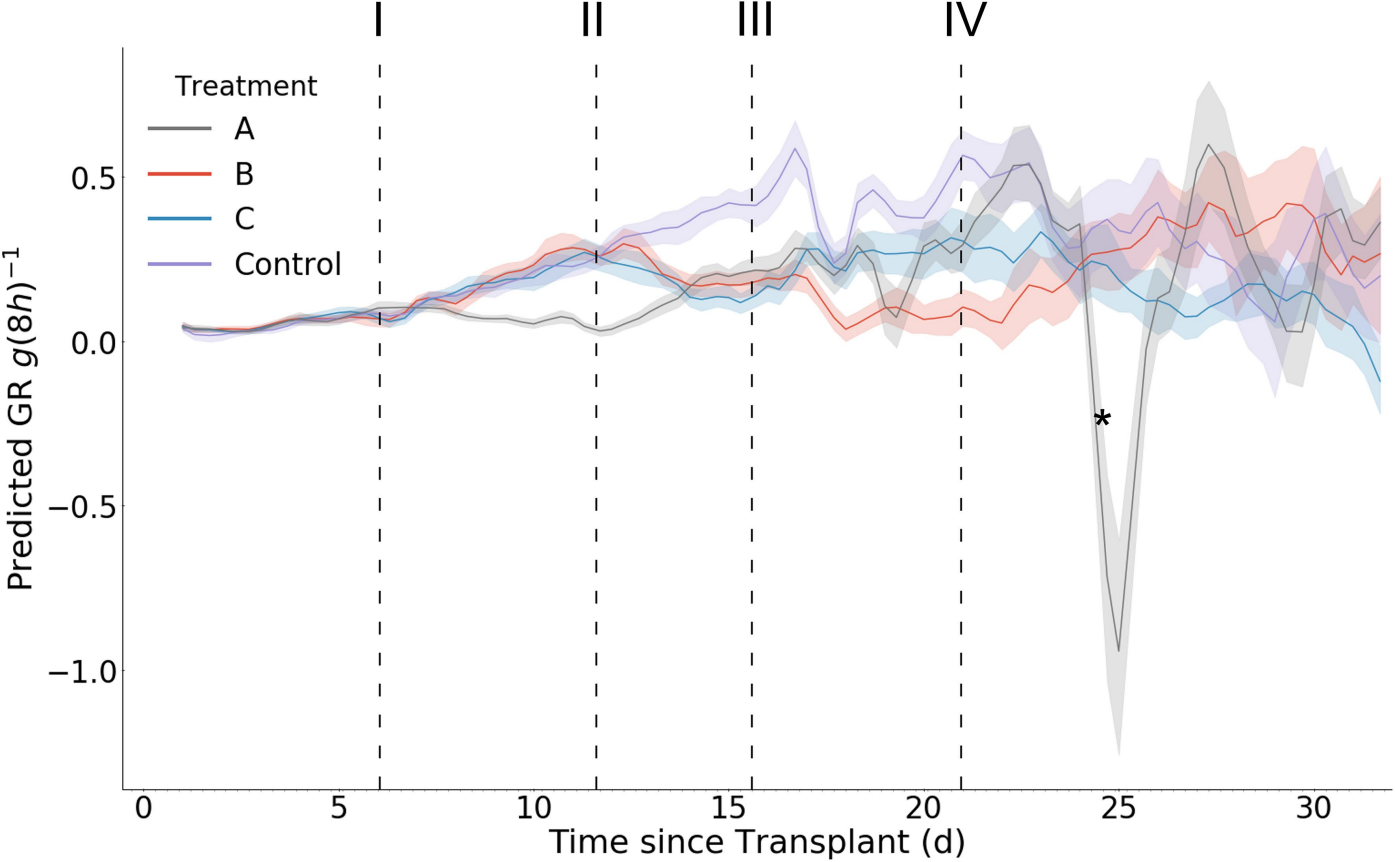
Mean per treatment predicted growth rate with 95% confidence interval. Y axis units are g (8 h)^−1^. Dark vertical lines and roman numerals indicate when treatments were applied. (I) Treatment A begins to receive pure water, (II) Treatment A begins to receive Hoagland solution and Treatments B and C begin to receive water, (III) Treatment C begins to receive Hoagland solution, and (IV) Treatment B begins to receive Hoagland solution and Treatment C begins to receive water. An asterisk marks where wilting in Treatment A was visually noticed in the image data (day 24.3).

**Figure 11:**
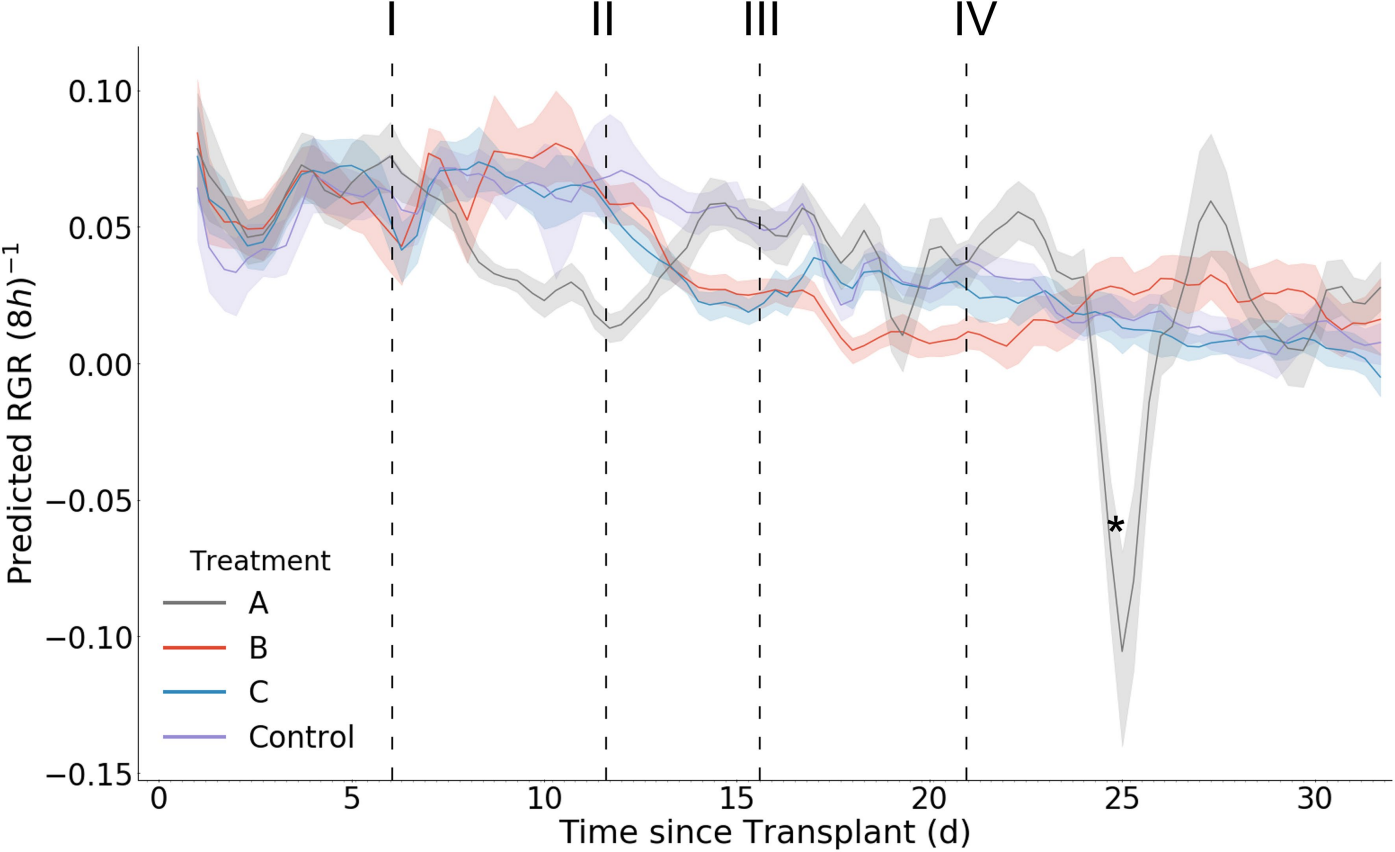
Mean per treatment predicted relative growth rate with 95% confidence interval. Y axis units are in (8 h)^−1^. Dark vertical lines and roman numerals indicate when treatments were applied. (I) Treatment A begins to receive pure water, (II) Treatment A begins to receive Hoagland solution and Treatments B and C begin to receive water, (III) Treatment C begins to receive Hoagland solution, and (IV) Treatment B begins to receive Hoagland solution and Treatment C begins to receive water. An asterisk marks where wilting in Treatment A was visually noticed in the image data (day 24.3).

In contrast with the model predicted biomass accumulation, the derived growth metrics GR and RGR exhibit significantly more daily fluctuation (Figures 10, 11). A result of this variation is the potential for mean separation between treatments that are experiencing identical growing conditions. Because of this, a simple difference in treatment mean GR or RGR is not a suitable metric for stress detection. Instead, we propose a criterion of two consecutive differences in GR or RGR means to indicate a significant change in growth dynamics between treatments. Stress response detection based on this criterion for GR is indicated at day 8.7 for treatment A compared with treatments B, C, and D; day 13.7 for treatments B and C compared with treatment D (control), and day 18.3 for treatment B compared with treatment C. Stress response detection for RGR is indicated at day 8.7 for treatment A compared with treatments B, C, and D; day 13.3 for treatments B and C compared with treatment D (control), and day 17.7 for treatment B compared with treatment C (Table 2).

**Table 2:** A summary of time until statistically significant treatment mean metric differences are observed for each stress event.

Metric	Stress event response time		
	Day 6	Day 11.7	Day 15.7
Biomass	3.3 d	3 d	5 d
GR	2.7 d	2 d	2.3 d
RGR	2.7 d	1.7 d	1.7 d

The stress events occurred on days 6, 11.7, and 15.7, and the times indicated for each metric are the number of days from each event until the time of data capture indicating treatment separation as provided by Tukey HSD test.

## Discussion

In this study we present a powerful technique for accurate non-destructive plant biomass prediction in visually occluded environments. While direct performance comparisons with prior studies are difficult due to a general lack of benchmark datasets for plant biomass estimation, we believe that our method represents state-of-the-art for the proximal non-destructive individual biomass estimation task. Moreover, we show that deep learning models can learn individual plant traits under heavy occlusion without explicit localizing annotations. The ability for deep learning models to implicitly learn occlusion lowers data labelling costs and allows for the potential to solve complicated tasks in agriculture that are challenging to solve *via* hand engineered computer vision algorithms. As data quantity can be large in agricultural systems, our method’s reliance on relatively inexpensive supervision in the form of biomass measurements allows for scalability that likely will only improve prediction accuracy. Finally, we have shown that it is possible to achieve high predictive performance on plant biomass estimation solely from color data acquired *via* readily available and low cost RGB sensors.

### Comparison With Prior Non-destructive Lettuce Biomass Estimation Efforts

The results presented here demonstrate a substantial advancement in our capacity to non-destructively estimate individual plant biomass under occluded growth conditions. In comparison with the results obtained by Mortensen et al. (2018), our model demonstrates substantially lower relative error at 7.3% compared to 40%. While the cropping system is not identical between this study and that of Mortensen et al. (2018), our work maintains lower relative error even under much greater occlusion and planting density. It should be noted that while the author’s field-based data acquisition system allowed for good control of lighting conditions, the scene background conditions are busier than our indoor acquisition system due to irregular soil and weed presence. Similarly, to our work, the authors’ method relies on images taken above the plant to be estimated. However, their method additionally requires the xy-coordinates of the stem emergence point to be known, as well as segmentation masks for validation of the underlying segmentation algorithm used to generate the fresh weight predictors. While our method relies on a far larger dataset, each data annotation is less expensive as we require only a single measured fresh weight value per plant.

In another study, Zhang et al. (2020) used a convolutional neural network approach to single plant biomass estimation but did so only on isolated plants lacking occlusion. While an RGBD camera was also used in their work, depth data was used only for an initial preprocessing segmentation step and not as model input. Despite a lack of occlusion from neighboring plants, their segmented RGB-only model appears to underperform both our RGB and RGBD models through visual comparison of the predicted versus observed scatter plots. However, a quantitative comparison between our studies is difficult to make due to missing information on the normalizing unit in the normalized RMSE performance metric used by the authors. If we do indeed achieve higher performance, we speculate that this is due to the use of a larger training dataset along with a DCNN regression architecture that leverages a pre-trained ResNet backbone.

### Learned Model Features and Generalizability

Perhaps one of the more powerful aspects of our modeling approach is the ability to implicitly learn to localize the center plant during training without explicit annotation. This localization is evident in the visualizations of feature map activations provided by GradCAM in both best and worst case prediction examples (Figure 8). Similar visualizations provided by GradCAM of the depth modality also show similar trends between the best and worst case predictions, including strong localization. However, it is evident that model starts to fail when the center plant is of lesser size than its neighbors. This is likely a result of two conditions: increased occlusion under these conditions and overfitting to the training dataset. In particular, the latter condition is due to the uniform nature of the cultivation practices used to generate the dataset, which resulted in scenes containing neighboring plants that generally (but not always) are of similar size and mass. Therefore, our model has likely learned to estimate center plant mass while also overfitting to some degree to common scene features of the training data distribution. Our plant monitoring experiment further supports this, as those scenes contained neighboring plants with significant variation in size and mass. The model’s ability to capture, in aggregate, expected changes in growth under such conditions suggests that it has learned to predict center plant mass. The model could be made more robust to variable sized plants by training with a more heterogeneous dataset; a hypothesis which warrants further investigation in future studies.

Another notable phenomenon is the relative lack of contribution of the depth data modality to model predictive performance. It is possible that our use of depth data is suboptimal for model learning. For instance, the pre-trained weights are tuned for feature extraction of color, and not depth, images. To allow for the use of pre-trained weights we map the depth data to a 3-channel color image, allowing the model to directly use its pre-trained feature extraction capabilities on the depth data. This mapping scheme imbues color data with a direct relationship to absolute distance from the sensor (e.g., red is farthest away while blue closest). While this does allow the model to learn biomass estimation to some extent, it is unknown whether it has maintained a strong understanding of absolute scene size and shape after this mapping due to the pre-training of the initial convolutional layer. Additionally, as our image capture height is fixed, the real world area represented by each pixel is essentially constant, allowing relative plant size to be estimated in the scene *via* RGB only. It may be the case that had image acquisition height been variable, depth data may have had an increased importance and outperformed color as the relative size of plants in each scene could no longer be directly tied to their absolute size in world space. We suspect that a modified architecture that can more directly utilize depth data would outperform, such as models that utilize 3D representations of the scene such as meshes or point clouds (Qi et al., 2017).

### Model Predictions for Plant Growth Monitoring

We have shown that our model is able to predict individual plant biomass with enough accuracy to capture the effects of nutrient stress on biomass accumulation within 2 days, even when those effects are applied with spatial heterogeneity. This illustrates model robustness to heterogenous plant size in the scene despite having been trained on more homogenous conditions. We further validate the response of the model by showing through hand collected measurement that our treatments had real effect on plant size, and by extension, in biomass accumulation (Supplementary Figure S2). However, it is difficult to quantify the true accuracy of the biomass, GR, and RGR calculations, as we have no ground truth for each treatment. Further, there are sources of variation in the response of each plant to the treatments, such as genotypic and block effects, which are not easily separable from model prediction error when examining the data in aggregate. Potential sources of block effects in include variations in lighting intensity, as well cross treatment contamination from plant roots which extend into the common drainage channels of the hydroponic system. As such, we do not have an exact understanding of the how our model’s predicted stress induction response time differs from the true stress induction response time.

While we do not know the true model biomass prediction error for this experiment, we do know that larger prediction errors would likely lead to a longer time before significant treatment effects can be determined by the Tukey HSD test. In a soilless top-fed hydroponic system such as ours, we would expect a significant change in fertigation solution (such as our nutrient stress treatments) to result in a close to immediate change to RGR. This is due to both the lack of cation exchange capacity of the stone wool medium and the ratio of medium volume to irrigation volume, likely resulting in a rapid change in concentration of nutrient ions in the root zone (Silber, 2008). Without access to nutrient ions, the plant’s biomass accumulation rate slows over time as it must rely only upon existing nutrient stores in its tissues to support cell growth and function. Our model required between 1.7 and 2.7 days to determine a significant treatment effect *via* RGR, which we believe is consistent with expected RGR of typical lettuce cultivars, the expected effect of complete nutrient deprivation on RGR, and our originally published model test error of 7.3% (Holsteijn, 1980).

The predicted RGR curves generally follow the expected shape as previously seen in the literature (Holsteijn, 1980). RGR decreases with time for all treatments as well as the control. During stress induction the RGR decreases, and then increases during stress reduction (Figure 11). An interesting phenomenon can be seen after stress reduction: a treatment’s RGR may rejoin that of the control provided no additional stress is introduced or sustained (Figure 11). The variance of the predictions within each treatment increases with time, as evidenced by larger mean confidence intervals. This is consistent with the expected model error as determined during our model validation. It should be noted that the ability to detect significant treatment effects was maintained beyond day 17, at which point plants of up to 15 g existed and contained significant occlusion. In addition to predicting RGR response between the comparable treatments *via* analysis of variance, by examining the graphs of RGR, GR, and biomass over time we can see that the effects of stress reduction (Treatment A on day 12) and stress induction (Treatment C on day 21) that occurred without a directly comparable treatment (Figures 9–11). The effect of Treatment C’s day 21 stress induction can be seen through day 32, revealing that our model maintains general accuracy even when neighboring plants (in particular, Treatment D) are large (Figure 9).

An unintended irrigation malfunction caused some individuals (approximately 15) within treatment A to experience water stress starting at approximately day 24. While we do not know exactly when water stress began for those individuals, it becomes visually noticeable in the image data at day 24.7 (Supplementary Figure S1). A reduction in predicted biomass is observed during this water stress period (Figure 9). That our model indicates a sudden change in biomass may practically be quite valuable and supports its sensitivity to changes in growth rate. While reduced predicted biomass is consistent with reduced plant water content under drought conditions, we exercise caution in concluding that our model can accurately extrapolate to individuals that are experiencing less than well-watered conditions, as such conditions are not represented in either the training or test datasets used to create and evaluate the model. This does leave open the possibility, however, that our model can serve as an indicator of crop response to environmental parameters that result in morphological change beyond biomass accumulation. Such sensitivity also allows for the future possibility of fine-tuning the model with data that includes plants experiencing wilting or other stress conditions, furthering the use of the model as a non-destructive plant stress detector.

## Conclusion

Our work introduces a novel technique that applies proximal vision based plant trait estimation models to the problem of stress detection and growth monitoring with high spatial and temporal resolution over the entire lettuce cropping cycle. By utilizing highly accurate biomass estimation models, short-term plant-environment interactions within cropping systems can be better monitored and quantified. Our brief exploration of the response in biomass accumulation to nutrient stress is far from exhaustive. Improvements could result from implementations featuring models with lower prediction error and more frequent data acquisition. To further determine the utility and ability of such sensing methods at scale, an implementation into a commercial facility should be conducted. This would help answer questions such as which stresses are best predicted by short term changes in biomass accumulation, on what basis do we make comparison of individuals for the purpose of stress or abnormality detection, and where do these methods fail in real world settings.

## Data Availability Statement

The raw data supporting the conclusions of this article will be made available by the authors, without undue reservation. Code available at https://github.com/NicoBux/Plant-Biomass-Monitoring.

## Author Contributions

NB, ME, and JL: conceptualization, performance evaluation, visualization, and review and editing. NB: data collection, model generation and testing, and writing—original draft. All authors contributed to the article and approved the submitted version.

## Funding

This project was partly supported by USDA AI Institute for Next Generation Food Systems (AIFS), USDA award number 2020-67021-32855.

## Conflict of Interest

The authors declare that the research was conducted in the absence of any commercial or financial relationships that could be construed as a potential conflict of interest.

## Publisher’s Note

All claims expressed in this article are solely those of the authors and do not necessarily represent those of their affiliated organizations, or those of the publisher, the editors and the reviewers. Any product that may be evaluated in this article, or claim that may be made by its manufacturer, is not guaranteed or endorsed by the publisher.
