# Supplementary material for: Non-destructive Plant Biomass Monitoring With High Spatio-Temporal Resolution via Proximal RGB-D Imagery and End-to-End Deep Learning
Source: Front Plant Sci. 2022 Apr 13;13:758818. doi: 10.3389/fpls.2022.758818 (PMC9043900; doi:10.3389/fpls.2022.758818)
Supplement: Supplementary file 1 [file Data_Sheet_1.docx]

Supplementary Material


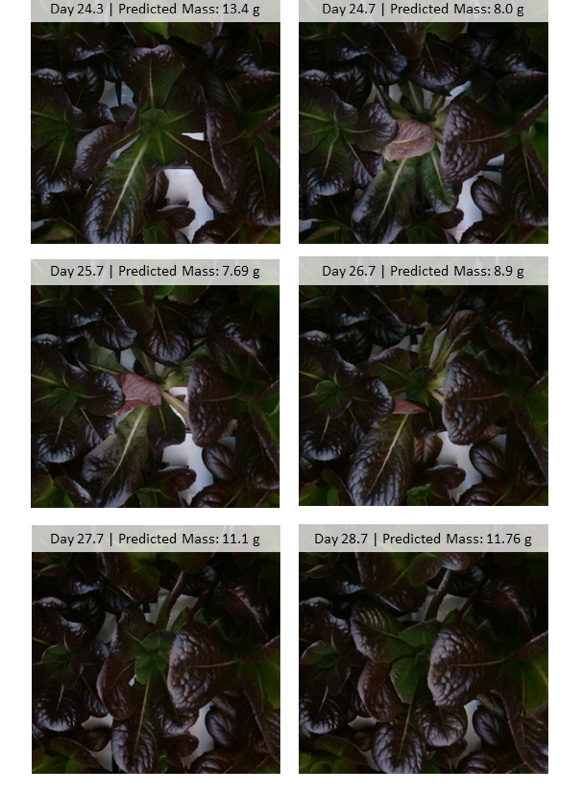


**Supplementary Figure 1**. Example individual experiencing water stress from treatment A, starting with one data collection event before noticeable water stress (day 24.3).


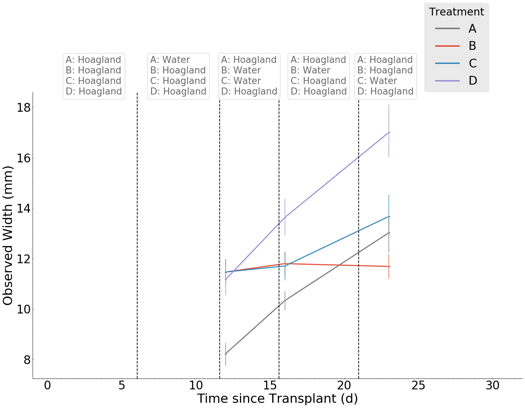

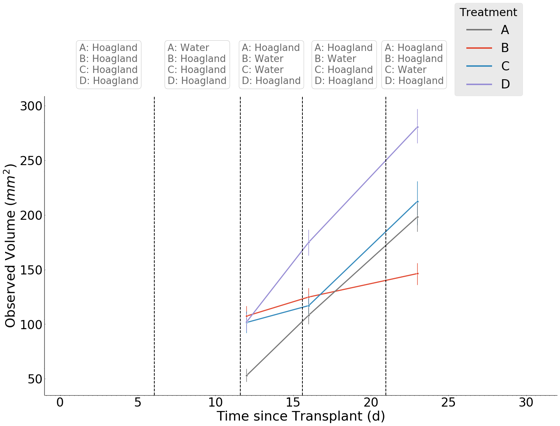

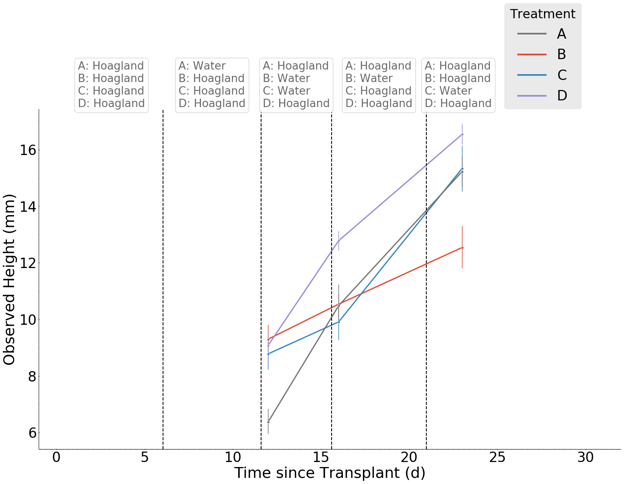


**Supplementary Figure 2**. Mean per treatment observed width, height, and volume with 95 % confidence interval indicated by vertical error bar. Y axis units are g(8h)^-1^. Dark vertical lines indicate when treatment schedules were applied. Annotated boxes indicate the current state of each treatment between the schedule intervals.
